# Extracting Technicians’ Skills for Human–Machine Collaboration in Aircraft Assembly

**DOI:** 10.3390/biomimetics8080604

**Published:** 2023-12-13

**Authors:** Yaling Tian, Ji Li, Junjie Dan, Yongsheng Shu, Chang Liu, Ruijie Li, Shiyong Liu

**Affiliations:** 1 School of Intelligent Manufacturing, Chengdu Technological University, Chengdu 611730, China; tyling1@cdtu.edu.cn (Y.T.); lrj1803322023@foxmail.com (R.L.); 2 School of Aeronautic Science and Engineering, Beihang University, Beijing 100191, China; 3 Chenghang Innovation Institute of Intelligent Aerocraft, Chengdu Aeronautic Polytechnic, Chengdu 610100, China; shu2116264965@gmail.com; 4 Chengdu Aircraft Industrial (Group) Co., Ltd., Chengdu 610091, China; lovejune1993@126.com (J.D.); liuchang001021@126.com (C.L.); loveliu1969@126.com (S.L.)

**Keywords:** aircraft assembly, human–machine collaborative, riveting, digital extraction, riveting experimental platform

## Abstract

Research on the efficiency and quality issues faced in aircraft assembly was conducted in this article. A new method of human–machine collaborative riveting was proposed, which combined the flexibility of manual collaboration with the precise control of automatic riveting. The research works include: (1) a theoretical model of pneumatic hammer riveting was established to clarify the principle and parameters of riveting process. (2) A smart bucking bar was designed to support the data collection and extraction of manual collaborative riveting process. (3) An automatic riveting experimental platform was designed to test the automatic riveting process incorporating the extracted manual riveting process parameters, and further an optimization strategy was proposed for the automatic riveting process. (4) A human–machine collaborative riveting experimental platform was developed to conduct the verification work. Through the theoretical analysis, experimental research, system scheme design, and process parameters optimization, the application and verification of human–machine collaborative assembly technology have been achieved. This technology is expected to be comprehensively promoted in the field of aircraft manufacturing, and for breaking through the current difficulties of low production efficiency and poor assembly quality control.

## 1. Introduction

Riveting is the most important means of aircraft assembly. Due to the complex structure of aircraft, various types of models and special functional areas, manual collaborative riveting where operators work in pairs (P-P mode) is the most suitable assembly mode. In the case of manual collaborative riveting, operators A and B are required to hold the bucking bar and hammer the rivet with rivet gun, respectively. And the formation of a rivet is formed by reciprocating riveting. This riveting process requires continuous observation, communication, collaboration, and riveting control. Due to severe noises and sight limitations on site, it is difficult to ensure the quality of manual riveting and connection. In the aspect of improving efficiency, it is popular to apply automatic riveting technology. The automatic riveting relies on special automatic riveting equipment, which requires large and open space. At present, automatic riveting machines are mainly applied in passenger plane assembly or large and open structure assembly. Their application is limited [1]. The most significant advantage of manual collaborative riveting is that it fully utilizes the high-flexibility of manual riveting to adapt to various complex structures, narrow spaces, and special assembly process. Its biggest drawback is control chaos: (1) there are two operators/controllers on site and no clear master–slave relationship between them; (2) due to environmental noises and sight limitations on site, it is difficult to effectively transmit control information; (3) operators cannot control the time accurately with the rapid dynamic deformation. The biggest advantage of automatic riveting is precise control and automatic execution, which helps to ensure quality and improve efficiency. Its biggest drawback lies in the large space required and poor flexibility of the equipment, which cannot adapt to the requirements of narrow spaces and complex structural environments.

A new technology for human–machine collaborative riveting (P-M mode) is proposed which combines the flexible merit of manual with the precise control of automated riveting, achieving improvements in efficiency, quality, flexibility and adaptability.

The literature study shows that manual operation is essential in aircraft riveting assembly. So, collaboration between human and machine with high-flexibility and automation has become a research hotspot in manufacturing industry [2]. The research hotpots in human–machine/robot include: human–machine task allocation [3,4], workflow analysis [5], digital twin driven human–machine/robot collaborative [6], and safety assembly [7]. At present, there is no research on the application of human–machine collaborative riveting, and no report about extracting the operators’ skill and then use it in human–machine collaboration.

In principle, there are two forms of automatic riveting. One is the pressing riveting with professional equipment, whose working process and technological scheme is hard to be transferred to human–machine collaborative riveting [1]. Another one is pneumatic hammer riveting, combined with robots or mechanisms for automated riveting. Xi F. F. has studied the technology of using a robot with pneumatic hammer riveting in aircraft assembly in the literature, developed a robot pneumatic riveting system, and studied the sequence of riveting process in this system [1]. Li Y. W. established a pneumatic hammer riveting process model with a robot automatic riveting system and proposed a method to calculate the speed of the riveting hammer. The impact dynamics and elastic–plastic deformation theory are used to analyze the rivet forming process and the riveting time with specific riveting parameters is determined [8]. Guo S. built the robots automatic riveting system with four robotic arms to analyze vibration in the riveting process and calculate the most suitable working space [9]. Li Y. W. studied the riveting system with mobile robots and found that both the force frequency and the mobile platform position have strong influence on the robotic riveting performance in terms of alignment during operation [10]. The above researchers conducted research on the robot automatic pneumatic riveting system, including robot dynamics, riveting process sequence, riveting gun alignment, and theoretical analysis of the rivet forming process.

In order to analyze the parameters of the riveting process, researchers established riveting experimental platform to conduct theoretical, simulation, and experimental research on the riveting process. The vibration mechanism of the riveting process was studied in the literature of Cherng, J.G, and Thomas to reduce harm to humans [11,12]. Zhang S. conducted pneumatic riveting experiments by machines, technicians, and trainees to analyze the influencing factors of pneumatic riveting forming [13]. An experimental platform is built to verify the finite element simulation model in the work of Wang H. L. [14,15]. Johnson established a nonlinear dynamic physics model which takes complex double riveting impacts and operator preloads into account as the riveting operates on both front and rear of the fuselage, to guide the experimental measurement process. It is demonstrated that rivet quality assurance indicators can be used to identify incorrect riveting processes and evaluate the susceptibility of the future damage of the fuselage structure [16]. Ahn constructed a human–machine ergonomic evaluation system for pneumatic riveting guns used in commercial aircraft manufacturing processes to analyze the effects of riveting frequency, riveting force, riveting time, and other parameters on operators [17]. Bloxsom established a digital model of a pneumatic riveting process in MATLAB to analyze the movement and pressure states of the riveting hammer inside the riveting gun barrel. Three brands of riveting guns were used for experiments and model comparison, achieving high-consistency in both time and frequency domains [18]. Kadam established a numerical model of pneumatic riveting, which includes an air fluid model and a structural dynamics model. The numerical model was used for parameterization research to explore different vibration control technologies [19]. Jiang L. P. conducted a stress field analysis, residual stress analysis of the elastic rebound of rivets and aluminum plates, and found that the interference amount and the rivet pier head height could meet the requirement by control the hammering force [20]. Zhang K. F. proposed a mathematical model including elastic deformation, plastic deformation, and elastic rebound three stages and the model is verified by FEA simulation results in ABAQUS [21].

Using the literature, we have studied the stages of riveting forming process and have some guiding significance for the analysis of riveting parameters. However, there are two issues that have not been well studied:

(1) The skills and experiences of technicians in manual riveting processes were not studied, including, A. how do the influencing factors work? Such as the gestures of operators, the frequency of hammer, and the force of riveting. B. How to extract these feature and skills? C. How to express these features digitally? D. How to apply these features in human–machine riveting or automatic riveting?
(2) The traditional pneumatic riveting is conducted by two technicians. As sight obstruction, the deformation results of rivet pier height are present on the other side of the aircraft panel, the technician who held the riveting gun cannot observe and make the decision to stop riveting immediately. The riveting is a rapid dynamic process, even if the technician could view it, it is difficult to control it precisely. The control decision can be made by either of them, when a rivet reaches qualified forming quality during the riveting process. It is difficult to synchronize two technicians, leading to confusion in decision-making and affecting the quality of riveting.

This article studied the process of manual riveting and its influencing parameters, proposed process parameters scheme and methods for automatic riveting control. An automatic riveting experimental platform and a human–machine collaborative riveting experimental platform are developed. The main work includes: (1) establishing a theoretical model of pneumatic riveting to clarify riveting control parameters; (2) designing a smart bucking bar to collect and extract technicians’ skill data from manual riveting process; (3) designing an automatic riveting experimental platform to test the automatic riveting process incorporating the extracted manual riveting process parameters; (4) designing a human–machine collaborative experimental platform that takes humans as the decision-making subject with the experience parameters obtained from (1) and (2). (5) Experimental verification.

## 2. Pneumatic Riveting Modeling

The pneumatic riveting system is a collision system composed of a riveting gun, rivets, aluminum alloy sheets, and bucking bar [8]. The riveting gun of Qian Shao M0501 is used for experiments. The parameters of riveting gun are introduced in Table 1.

**Figure 1:**
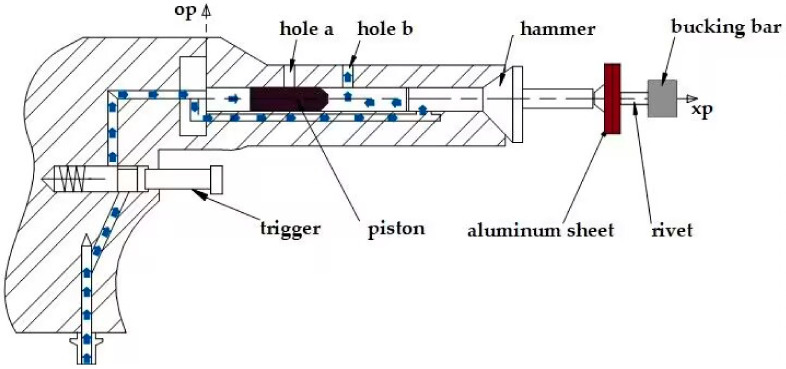
The working mechanism of pneumatic riveting gun.

The working mechanism of pneumatic riveting is shown in Figure 1. When the riveting gun is working, the compressed air enters the left and right sides of the piston in the riveting gun barrel. The compressed air on the right side is discharged into the atmosphere through hole b and a pressure difference between the left and right is formed when the piston is at the left end. The piston moves to the right and collides with the riveting hammer and the impact energy acts on the rivet and causes its deformation. The piston rebounds and hole b is blocked when it hits the riveting hammer. A pressure difference is formed by discharging the air from the left side through hole a. Then the piston moves to the left under the action of impact rebound and pressure difference. The rivet is formed by recursive hammering.

A coordinate system $\left\{O_{P}X_{P}\right\}$ is established to describe the piston motion. The equation of piston motion is given here:

(1) $$m_{p}{\ddot{x}}_{p}=pS_{p}+\mathrm{sgn}(v_{p})um_{p}g,$$

where $m_{p},\;x_{p},\;S_{p},\;v_{p}$ are the mass, displacement, cross-sectional area and velocity of the piston, respectively, p represents the pressure acting on the piston, $\mathrm{sgn}(v_{p})$ is used to determine the direction of friction, u is the friction coefficient between the piston and the barrel, and g is the gravity acceleration.

The piston impacts the rivet hammer when the piston reaches the maximum stroke. According to the Conservation Law of Momentum and Kinetic Energy, the equation of the speed of the piston and rivet hammer after impact are given here:

(2) $$v_{p1}=\frac{(m_{p}-m_{h})v_{p0}}{m_{p}+m_{h}},$$

(3) $$v_{h}=\frac{2m_{p}v_{p0}}{m_{p}+m_{h}},$$

where $v_{h},\;m_{h}$ are the velocity and mass of the hammer in riveting gun. The impact energy acts on the rivet through the rivet hammer. Since the rivet and the hammer keep in contact during the riveting process, assuming that all the energy is converted into strain energy, regardless of the kinetic energy, there are:

(4) $$E_{i}=\frac{m_{p}v_{p}{}^{2}}{2}=\frac{m_{h}v_{h}{}^{2}}{2}=S_{r}L_{r}\int_{0}^{\varepsilon_{rx}}\sigma_{rx}d\varepsilon_{rx}$$

where $E_{i},\;S_{r},\;L_{r},\;\varepsilon_{rx},\;\sigma_{rx}$ are the kinetic energy, cross-sectional area, length, strain and stress of rivet.

Based on the above study, the pneumatic riveting gun is a key equipment in the human–machine collaborative assembly system. The single strain or deformation is determined by the initial velocity of the piston when collision. While the velocity is determined by p and friction. The sum of the strain is total amount of deformation, which determined by the number of impacts. The number of impacts is reflected by the riveting time and impact frequency. In manual operation, p is determined by the effective pressure which is affected by two factors: (1) the input air pressure of riveting gun; (2) the depth at which the operator presses the trigger. The first one is a controllable factor, while the second one is subjective and not controllable.

**Figure 2:**
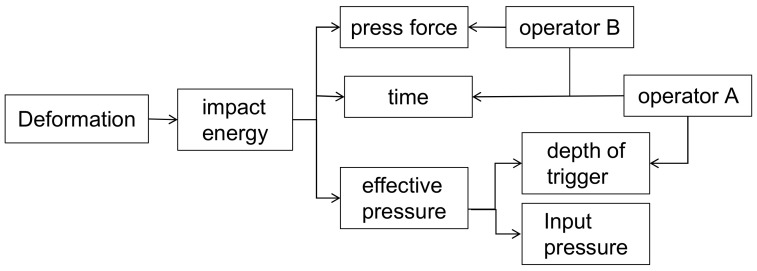
The relationship of control parameters.

The relationship of the control parameters is shown in Figure 2.

Based on the analysis, there are two key parameters which need to be determined to develop the subsequent human–machine collaborative control system. The first one is effective riveting pressure, the second one is the riveting time. While the effective pressure cannot be obtained directly. So, in the next part, a smart bucking bar is designed to collect data to analysis the effective riveting pressure indirectly.

## 3. Research on Manual Riveting

### 3.1. Smart Bucking Bar

**Figure 3:**
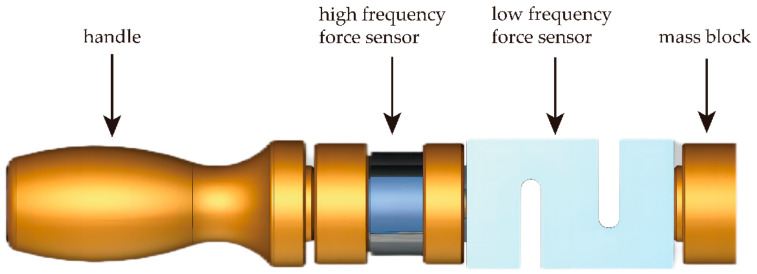
The structure of the smart bucking bar.

A smart bucking bar with data collection function is designed. It is equipped with a Dayang DYZ-101 column sensor with a response frequency of 10 kHz, which can effectively obtain the impact force signal during the riveting process. The riveting time, peak riveting force, number of impacts, and press force of riveting technicians can be obtained by the signal process. The structure of the bucking bar is shown in Figure 3.

### 3.2. Manual Riveting Experiment

**Figure 4:**
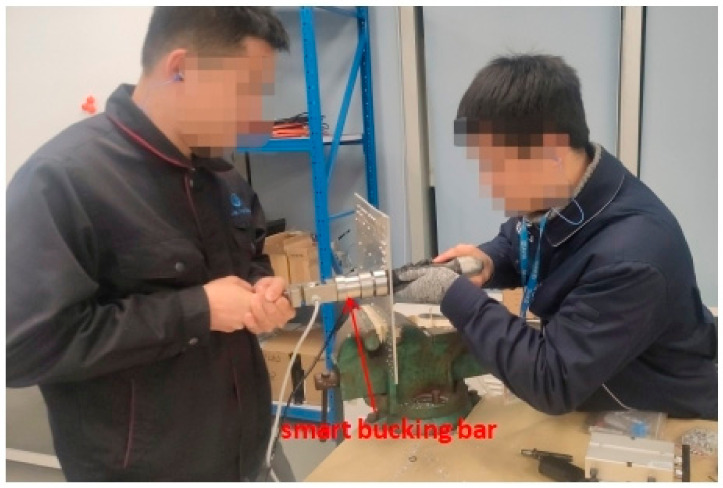
Manual riveting experiment with smart bucking bar.

The experiment is designed to extract the experience parameters of technicians. Using real operating conditions, with an input air pressure of 0.63 MPa for the riveting gun. The riveting time is determined by the technicians’ experience and the operation is stopped by visual judgment of the forming quality. The experimental operation of riveting technicians holding the smart bucking bar is shown in Figure 4.

### 3.3. Analysis of Experimental Data for Manual Riveting

**Figure 5:**
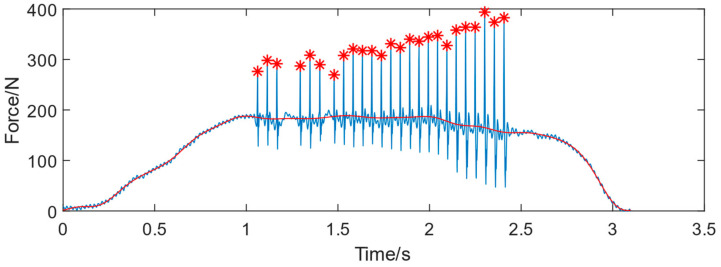
The signal process of manual riveting signal (the red asterisk means the peak force value of each impact, the blue line means the real-time riveting force, and the red line means the press force provided by the operator).

According to the analysis of experienced technicians’ riveting signals, it can be seen from Figure 5 that the riveting impact force mainly exists in the form of high-frequency fluctuations, while the manual press force is a low-frequency signal. Fourier transform is used to process the collected signal, and the low-frequency press force signal is the red curve shown in Figure 5. The press force signal starts from 0 and gradually increases and the entire riveting process fluctuates significantly with poor consistency. Figure 5 shows a riveting process with multiple impacts. The red asterisk means the peak signal of each impact during the riveting process, which is the main energy for riveting forming.

**Figure 6:**
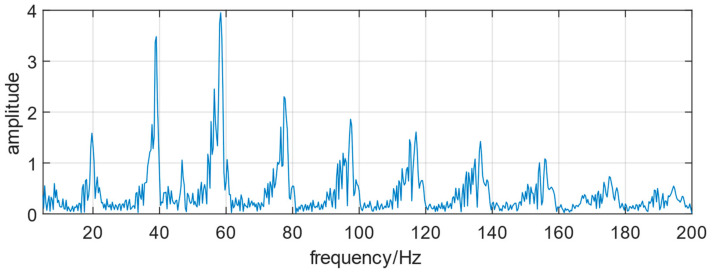
Amplitude frequency plot of manual riveting signal.

The amplitude frequency diagram of manual riveting is shown in Figure 6.

The range of press force, peak value of riveting force, riveting frequency, riveting time, and pier head height of nine sets manual riveting operations are shown in Table 2.

Based on the experimental data, the following conclusions are drawn:

(1) The peak impact force during manual riveting is unstable and varies with the depth of the riveting technicians pressing the trigger (effective input air pressure);
(2) The riveting time is distributed between 1.345 s and 1.9262 s, with a wide fluctuation range. The riveting time exceeds 1 s, resulting in low riveting efficiency;
(3) The number of riveting impacts is 25–31, and the frequency is lower than the rated frequency of 25 HZ;
(4) The press force is between 71.36 N and 253.62 N;
(5) The maximum range of peak riveting force is 291.44–436.79 N, and the minimum range of peak riveting force is 126.91–293.48 N;
(6) The bigger the press force and riveting force, the smaller the forming height of the rivet pier head;
(7) The pier head height is between 1.81 mm and 2.11 mm. All of them meet industry standards which is 1.6–2.5 mm, the average value is 1.98 mm, the standard deviation is 0.09 mm, and the forming height range is 0.3 mm.

## 4. Research on Process Parameters of Automatic Riveting

### 4.1. Design of an Automatic Riveting Experimental Platform

**Figure 7:**
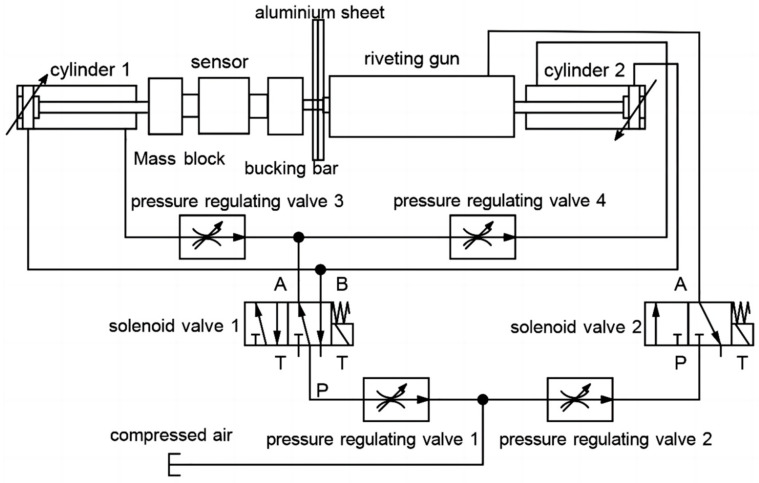
Block diagram of automatic riveting experimental platform.

We designed an automated hammer riveting experimental platform that requires, (1) automatic riveting function: holding a riveting gun, bucking bar, and aluminum plates to be riveted; (2) signal detection and collection function: detect and collect riveting force and press force; (3) riveting. The press force acted on the bucking bar and riveting gun, adopting pneumatic tightening rather than fixed riveting gun or bucking bar. The riveting gun and bucking bar is able to move in an axial direction based on actual force because of the pneumatic press. The aluminum plates to be riveted are fixed with fixtures, the bucking bar and riveting gun are supported in the vertical direction. The riveting force is collected by high-frequency sensors. The press force changes slowly and can be directly measured in non-working conditions. In working conditions, the press force is calculated by signal process and cannot be measured directly during the impact process. The block diagram of automatic riveting experimental platform is shown in Figure 7.

**Figure 8:**
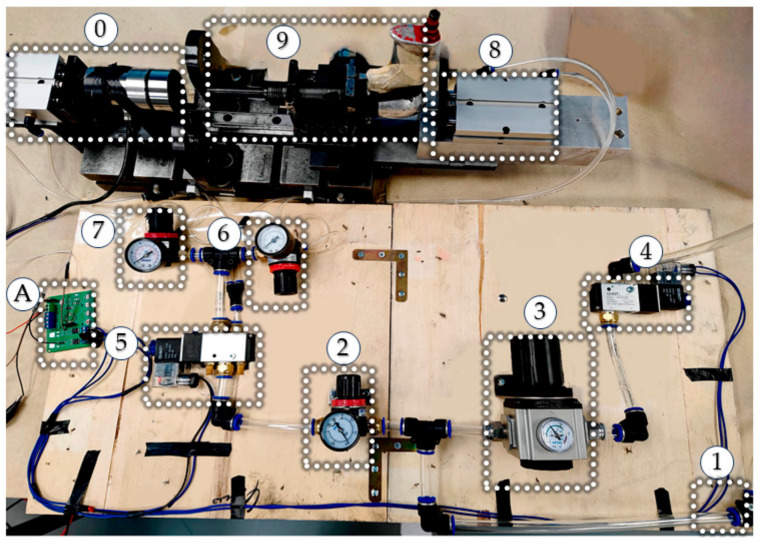
The automatic riveting experimental platform (0—intelligent bucking bar, 1—Compressed air, 2—Pressure regulating valve 1, 3—Pressure regulating valve 2, 4—solenoid valve 2, 5—solenoid valve 1, 6—pressure regulating valve 3, 7—pressure regulating valve 4, 8—cylinder 2, 9—riveting gun, A—control chip).

The automatic riveting experimental platform is shown in Figure 8.

### 4.2. Research on Process Parameters of Automatic Riveting

#### 4.2.1. Analysis of Input Pressure of Press Force

**Figure 9:**
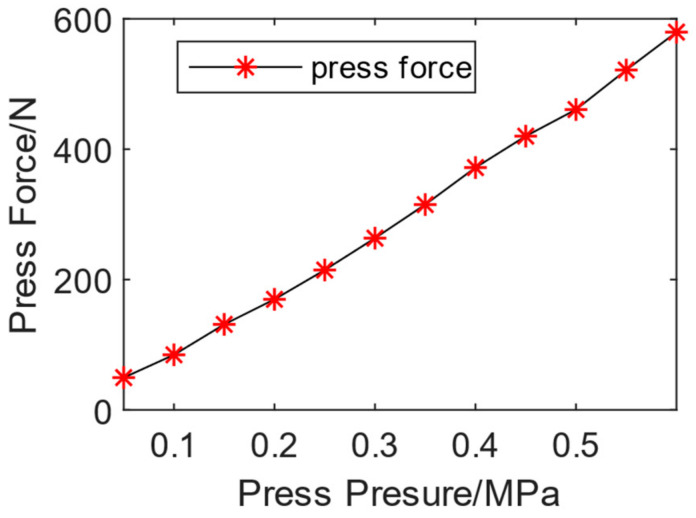
The relationship between press force and press pressure.

According to the extraction of manual riveting data, the press force is 71.36–253.62 N. The input air pressure of the press force is used as the control parameter, increasing gradually from 0.05 MPa to 0.6 MPa at intervals of 0.05 MPa to obtain the corresponding air pressure range of the manual press force range. The experiment collected press force data via static loading, and the average press force during the stable stage at each press pressure is shown in Figure 8. A pressure of 0.05 MPa corresponds to 49.52 N, and 0.6 MPa corresponds to 579.59 N. As the press pressure increased, the press force almost linearly increased. According to the experimental results, it can be concluded that controlling the range of press pressure between 0.1 MPa and 0.3 MPa can be consistent with the press pressure during manual riveting. The relationship between press force and press pressure is shown in Figure 9.

**Figure 10:**
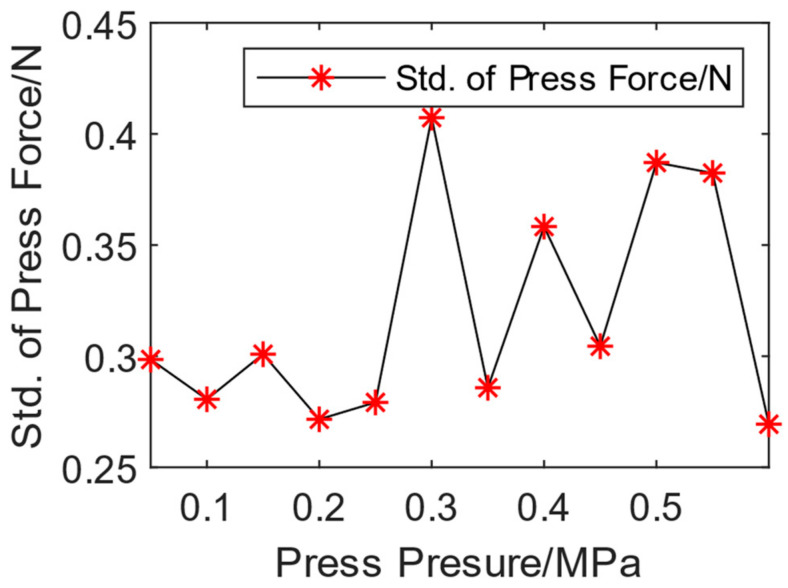
The standard deviation (Std.) of the press force at different press pressures.

The standard deviation of the press force under each air pressure is shown in Figure 10, which fluctuates between 0.25 and 0.41. The fluctuation range of the press force is little, and compared to manual press, the stability, consistency, and continuity of the press force generated by automatic mode are higher, which is easier for automatic riveting control.

#### 4.2.2. Analysis of the Air Pressure Range of Automatic Riveting Gun

**Figure 11:**
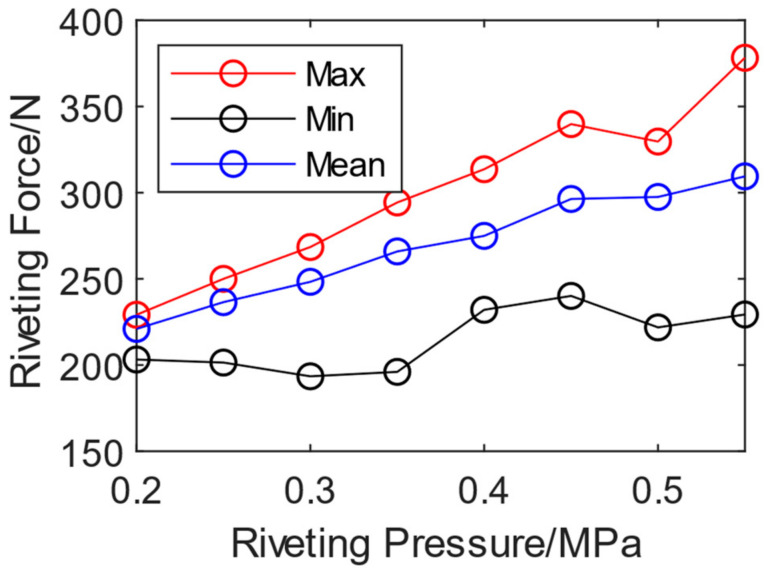
Relationship between riveting pressure and riveting force.

The average peak value of riveting impact force ranges from 236.47 N to 371.48 N. In automatic mode, the riveting force is controlled by the air pressure of the riveting gun. Therefore, experiments were performed to obtain the riveting force range as the corresponding riveting input air pressure range. Specifically, the input air pressure of the riveting gun was set to 0.2–0.6 MPa with intervals of 0.05 MPa increased. The riveting force is shown in Figure 11. The maximum and average variation trends of the peak riveting force are stable, gradually increasing with the increase of riveting air pressure. The maximum value corresponding to 0.2 Mpa is 229.16 N, and 0.55 MPa reaches 378.26 N. The minimum peak value appears at the first impact because the riveting compressed air is turned on at the beginning of the riveting and not stable yet. So, there is no obvious linear relationship between the minimum peak values of riveting force and riveting pressure. To obtain the manual riveting force range, the riveting gun pressure can be set as 0.2 MPa to 0.6 Mpa.

#### 4.2.3. Analysis of Press Pressure and Pier Head Height

The press force is loaded statically. Based on the above analysis, the stress generated by the maximum press force is calculated by σ = F/s = 5.03 × 10^6^ Pa and is much smaller than the yield strength of the rivet, which is 2.65 × 10^8^ Pa, the press air pressure only leads to elastic deformation and has no direct action on riveting forming. When working in human–machine collaboration mode, to simplify the control scheme, it prefers a human pressing the bucking bar to a human riveting with a gun.

#### 4.2.4. Analysis of Riveting Air Pressure and Pier Head Height

**Figure 12:**
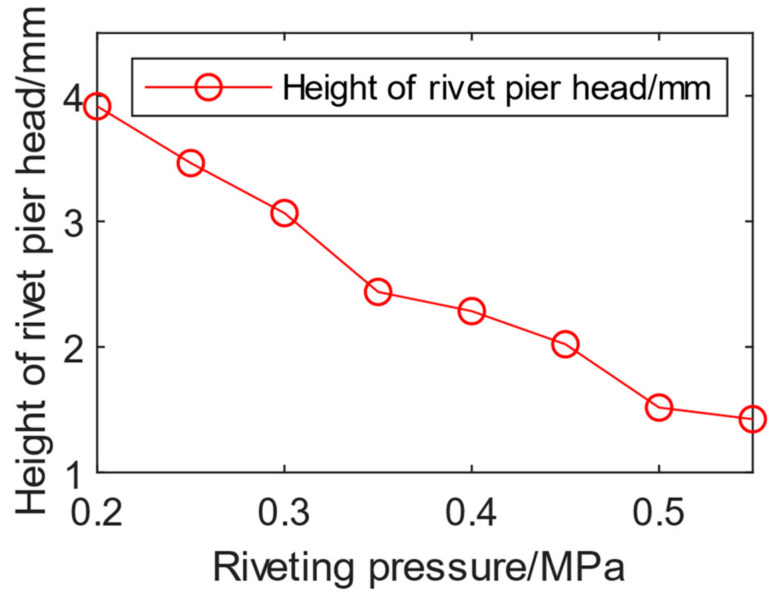
Relationship between riveting air pressure and pier head height.

Then the relationship between the riveting air pressure and the pier head height was analyzed. The riveting time was set to 1 s based on the time of manual riveting operation. The riveting air pressure was set as 0.2 MPa to 0.55 MPa. The riveting forming results corresponding to each riveting air pressure are shown in Figure 12. As the riveting air pressure increases, the height of the pier head gradually decreases, with 0.2 MPa corresponding to the pier head height of 3.92 mm and 0.55 MPa corresponding to the pier head height of 1.42 mm. The forming height of the pier head is linearly negative correlated with the riveting pressure.

### 4.3. Research on Human–Machine Collaborative Riveting Process Plan

The manual riveting process parameters was extracted to guide and optimize human–machine collaborative riveting parameters. The peak range of impact force, impact frequency, riveting time, and press force during the manual riveting process were studied. According to the analysis of experimental data, it can be seen that the press pressure range is set as 0.1–0.3 MPa, and the input pressure range of the riveting gun is 0.2–0.6 MPa. By controlling the appropriate riveting time, the required formation height can be achieved.

There are two ways of human–machine collaboration riveting:

(1) Technician holds the bucking bar (reverse riveting): The technician controls the start time of riveting and the riveting operation is conducted by the machine (or robot) with a set of parameters (riveting air pressure and riveting time);
(2) Technician holds the riveting gun (forward riveting): The technician controls the start time of riveting, and the riveting pressure, riveting time, and press pressure are set in the controlling procedure.

The riveting quality, efficiency, and energy consumption are considered when optimizing the process parameter. From experimental analysis, the higher the riveting pressure, the faster the forming process. While the shorter the time, the lower the control accuracy and the more difficult to ensure quality. So, the evaluation of process parameter schemes is a function of riveting air pressure, press air pressure, and riveting time, which can be written as follows:

(5) $$s=f(P_{r},P_{j},t_{r})$$

## 5. Design and Verification of Human–Machine Collaborative Riveting Experimental Platform

### 5.1. Human–Machine Collaborative Riveting Experimental Platform

The direct control parameters for the riveting process were analyzed before, including the riveting input air pressure, riveting time, and press pressure (press force). In the human–machine collaborative riveting system, the riveting gun is tightened in the axial direction by cylinder 1 through controlling solenoid valve 1, and the tightening pressure is adjusted via pressure regulating valve 3. The axial direction of the riveting gun can be moved which simulates human operation. The pressure regulating valve 2 regulates the input air pressure of the riveting gun, and the solenoid valve 2 controls the on–off of the input air pressure of the riveting gun to achieve the start and stop of riveting. The technician holds the smart bucking bar to press the tail of the rivet.

During the riveting process, the technician only needs to control the press force of the smart bucking bar. All other parameters are set before operation, and the on–off time of the solenoid valve is controlled by the program in control chip. It is effective to fix the decision-making issue.

**Figure 13:**
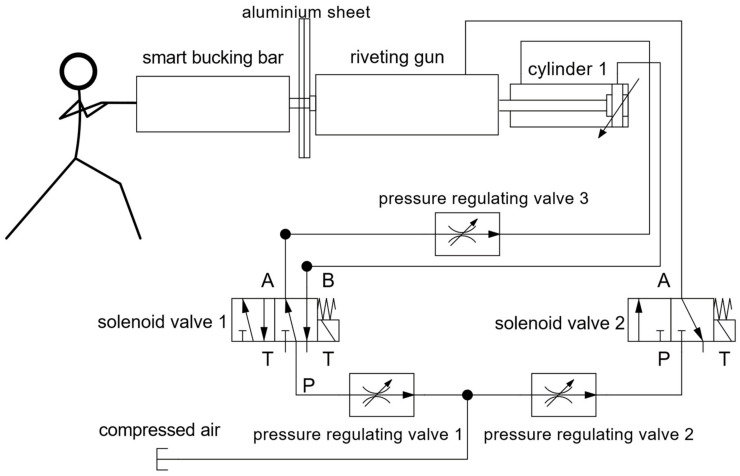
Block diagram of human–machine collaborative riveting experimental platform.

The working principle of the platform is shown in the following Figure 13.

**Figure 14:**
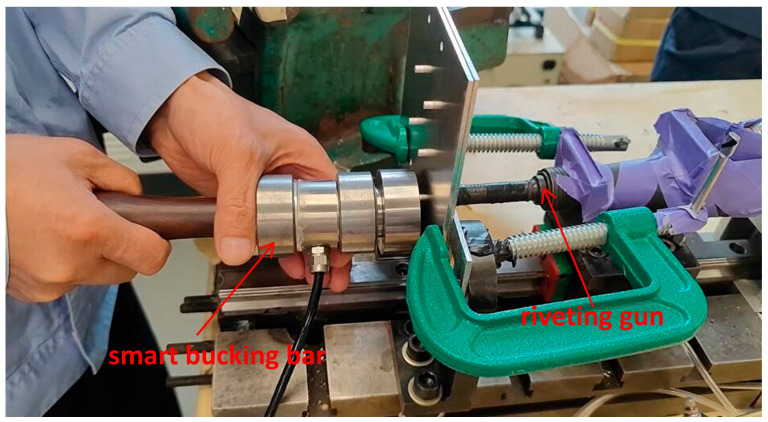
Human–machine collaborative riveting experiment.

The human–machine collaborative riveting experiment is shown in the following Figure 14.

### 5.2. Optimization and Selection of Human–Machine Cooperation Parameters

The parameters’ value of riveting input air pressure, riveting time, and press pressure for specific specifications of rivets and aluminum sheets were optimized based on the experience of previous experiments. Specifically, (1) for riveting input air, the qualified riveting pier head height can be formed with input air pressure between 0.2 and 0.63 MPa, approximately. However, the lower the riveting air pressure, the smaller the single impact energy, the longer the riveting operation time, and the lower the efficiency. The higher the riveting pressure, the greater the single impact energy, the less impact times required for forming, but the greater the control error. Therefore, in the selection of riveting pressure, it is necessary to balance both efficiency and control error. In this article, 0.425 MPa is selected to guarantee stable control with satisfactory efficiency. (2) Riveting time is a parameter influenced by the riveting air pressure. Once the riveting pressure is determined, the time can be obtained via the experiments. A time of 1 s is set in this article based on the experiments. (3) From the results of manual riveting experiments, press pressure is required to distribute between 0.1 MPa and 0.3 MPa. In addition, the press pressure should be less than riveting pressure to ensure that the rivet not be pushed out.

### 5.3. Human–Machine Collaborative Riveting Experiment

In the experiment, the technician who holds the bucking bar is the main control body. Two sets experiments were conducted here, with riveting time and riveting pressure parameters. The riveting experiments with a target pier head height between 1.6 and 2.5 mm, approximately, were conducted using an initial manual riveting pressure of 0.63 MPa and an optimized pressure of 0.425 MPa, respectively. The riveting experimental parameters are shown in the Table 3.

**Figure 15:**
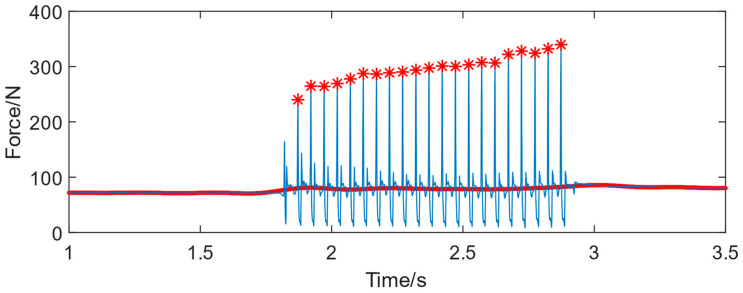
The riveting signal of human–machine collaborative riveting with 0.425 MPa. (the red asterisk means the peak force value of each impact, the blue line means the real-time riveting force, and the red line means the press force provided by the operator).

The riveting force signal of human–machine collaborative riveting with 0.425 MPa is shown in Figure 15. compared with the manual operating (Figure 5), the signal consistency is much better. The impacts of the riveting are controlled via the machine, with no discontinuity situations. The press force to the bucking bar is more stable than the manual mode. The experimental results are shown in Table 4.

**Figure 16:**
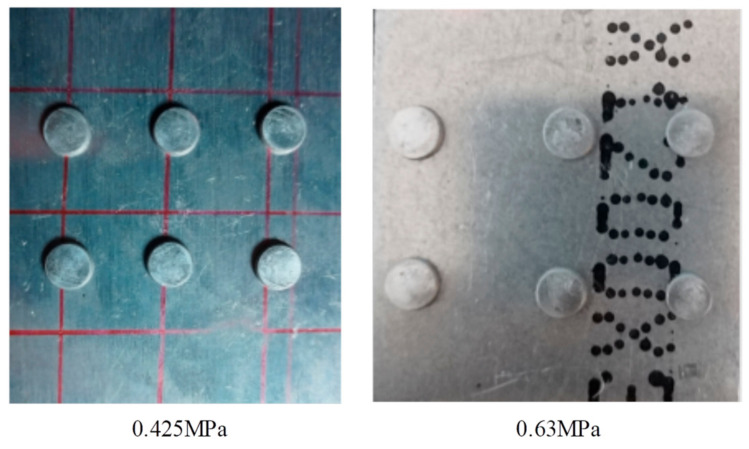
Human–machine collaboration riveting experimental specimens.

From the experimental data, it can be seen that the optimized riveting pressure of 0.425 Mpa has a higher consistency in the height of the pier head, with a range of 0.1 mm. However, with an initial pressure of 0.63 Mpa, the range of pier head height is 0.32 mm, with a wide distribution range and poor consistency. Human–machine collaboration riveting experimental specimens is shown in Figure 16.

## 6. Conclusions

This article studied new technologies and methods in the fields of manual collaborative riveting, automatic riveting, and human–machine collaborative riveting. The skills and experiences of technicians were extracted and integrated in the designed human–machine collaborative platform. Based on the studies and experiments, the following conclusion were obtained:

(1) The extraction and digitization of manual collaborative riveting process parameters is valuable. These parameters can be used in auto mode and human–machine mode to obtain qualified riveting results.
(2) The riveting quality is controlled by program automatically in the designed human–machine platform. The technician only needs to pay attention to the press force acting on the smart bucking bar which effectively avoids the decision-making confusion issue.
(3) Compared to the manual riveting, human–machine mode proposed in this article has more stable force signal and precise control in riveting process which results in higher consistency of riveting results.
(4) A qualitative analysis of the relationship among riveting pressure, press pressure, riveting time and riveting quality is conducted in this article which provide support for optimizing the selection of parameters. The next step can be quantitative analysis.
(5) A new riveting mode human–machine collaborative riveting is proposed for this field. At the same time, the digital extraction technology of technicians’ experiences proposed in this article can support digital monitoring of the pneumatic riveting process, which is helpful for monitoring the entire manufacturing process.

In the next study, we plan to create a matching relationship among the press force, the riveting pressure and the riveting time (number of impacts) to describe a high-quality riveting model. Then, the press force will be extracted at the beginning (the first 30%) stage of the continuous impact to obtain the press force characteristics of the operator. Finally, the system will automatically match the riveting pressure and riveting time for the remaining impact process to guarantee high-quality riveting results.

The adaptive riveting technology that automatically matches operators with appropriate riveting parameters is expected to be promoted and applied in the aviation industry.

## Figures and Tables

**Table 1:** The parameters of rivet gun.

Riveting Ability	Impact Energy	Impact Frequency	Gas Consumption	Working Pressure	Tool Weight
Al 5 mm	3.9 J	25 HZ	4.16 L/s	6.3 kg	1.18 kg

**Table 2:** Nine sets of manual riveting data statistics.

No.	Time/s	The Peak of Riveting Force				Press Force/N	No. of Impacts	Head Height/mm
		Max./N	Min./N	Mean./N	Std.			
1	1.92	436.79	250.80	371.48	45.27	213.30	26	2.01
2	1.78	413.92	278.91	337.11	35.62	201.40	26	2.11
3	1.79	410.43	293.48	362.82	34.66	253.62	30	1.81
4	1.65	317.92	233.42	283.60	18.08	103.00	30	2.02
5	1.35	393.71	269.50	327.10	33.36	186.60	25	2.02
6	1.64	315.91	203.36	252.94	33.39	98.62	26	2.04
7	1.55	333.45	209.84	265.23	39.83	93.90	26	2.00
8	1.89	291.44	126.91	236.47	42.95	71.36	31	1.89
9	1.93	366.76	210.23	291.51	43.51	137.90	30	1.91

**Table 3:** The experimental parameters of human–machine riveting.

Item	No. 1	No. 2
Riveting pressure	0.425 MPa	0.63 MPa
Riveting time	1.0 s	0.5 s
Rivet material	2A10	2A10
Rivet No.	HB6298	HB6298
Aluminum plate 1	2A12	2A12
Aluminum plate 2	2A12	2A12
Press Pressure	0.1–0.3 MPa	0.1–0.3 MPa

**Table 4:** The experimental statistical results (units: mm).

Riveting Pressure/Mpa	1	2	3	4	5	6	7	8	Std.	Range
0.425	2.11	2.10	2.19	2.20	2.14	2.12	2.18	2.17	0.04	0.1
0.63	2.00	2.01	2.11	2.04	2.32	2.31	2.30	2.25	0.53	0.32

## Data Availability

Data is unavailable due to privacy.
